# Hydropneumothorax Revealing a Pneumoblastoma in Children

**DOI:** 10.1155/2020/8879661

**Published:** 2020-09-09

**Authors:** Karima El Fakiri, Ghizlane Draiss, Noureddine Rada, Mohammed Bouskraoui, Abderrachid Hamdaoui, Mohamed Oulad Saiad

**Affiliations:** ^1^Pediatric A Department, Pediatric Pulmonology Unit, Mother and Child Hospital, University Hospital Mohammed VI Marrakesh, Marrakesh, Morocco; ^2^Pathology Laboratory Zohor El Hadika El Kobra Marrakesh, Marrakesh, Morocco; ^3^Pediatric Surgery Department, Mother and Child Hospital, University Hospital Mohammed VI Marrakesh, Marrakesh, Morocco

## Abstract

Pneumoblastoma is a rare primary childhood tumor. We report the observation of an infant aged 2 years and 8 months who presented with dry cough and dyspnea. The physical examination found mixed pleural effusion syndrome on the right. The chest X-ray revealed a right pneumothorax. Biology has shown leukocytosis at 16,000/mm^3^. The CT scan revealed parenchymal air cystic lesions affecting the outer segment of the middle lobe mimicking a pulmonary malformation. Thoracic drainage brought back 100 ml of the fluid. Two months later, when a pyopneumothorax appeared, a medium lobectomy was performed. Pathological study specimen showed a high-grade type II pneumoblastoma The extension assessment identified a secondary hepatic location. Chemotherapy has been indicated. This observation illustrates the diagnosis challenge of pneumoblastoma in children.

## 1. Introduction

Pneumoblastoma (PB) is an extremely rare primary malignant tumor in children. It represents 0.25 to 0.5% of all lung tumors [1] with a very serious prognosis. Classically, there are three types of PB [2]: type I is a purely cystic, bullous lesion, type II combines solid and cystic plaques, and type III is exclusively solid. The clinical features of PB are usually nonspecific encompassing pneumothorax or respiratory distress [3] sometimes leading to delayed diagnosis. X-ray images are often confused with those of a congenital lung defect. Metastasis from PB can affect the central nervous system, bone, and liver. Treatment is based on surgery and neoadjuvant chemotherapy. Here, we report an unusual observation after the consent of the parents of a hydropneumothorax revealing pneumoblastoma in a girl mimicking pulmonary malformation.

## 2. Case Report

**Figure 1:**
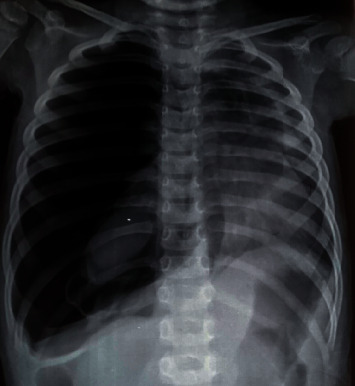
Chest X-ray showing the presence of a right-sided spontaneous pneumothorax with a midline shift to the left.

**Figure 2:**
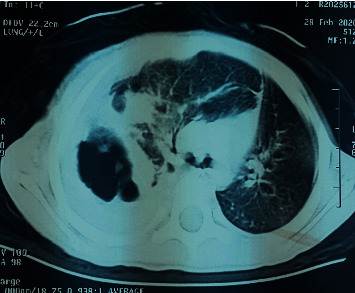
CT scan showing overdistension of the right chest with parenchymal air cystic lesions involving the external segment of the middle lobe.

**Figure 3:**
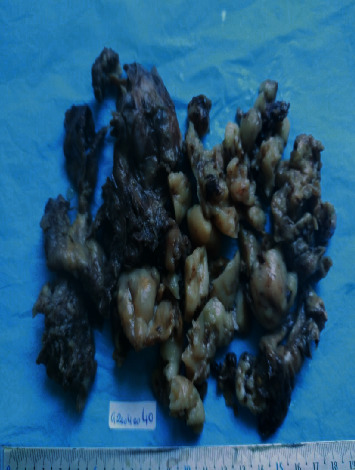
Multiple resected fragments with a gelatinous component with necrotic fragments.

**Figure 4:**
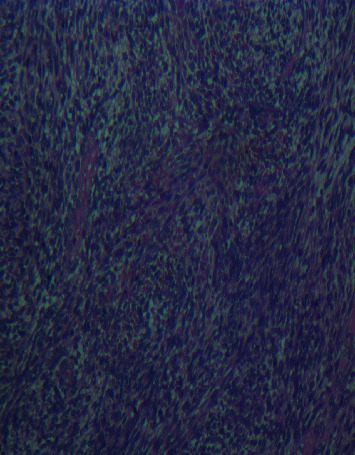
Photograph of pneumoblastoma type II showing malignant mesenchymal cell proliferation.

**Figure 5:**
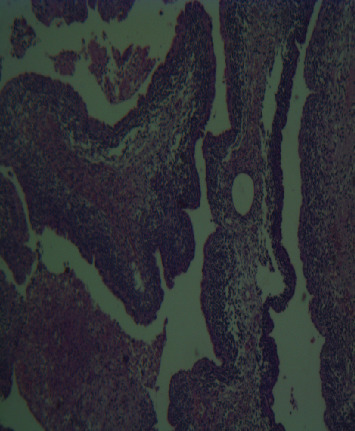
Photograph of pneumoblastoma type II showing a multicystic structure with the epithelial component.

We report a case of a 2-year-8-month-old female without parental consanguinity. She was vaccinated up to date according to the national immunization program. No known recent tuberculosis or personal atopy and no family history of tumors were found. She was referred for a dry cough, becoming wet, dyspnea, and chest pain with fever. The clinical examination found a conscious patient with fever at 38.5°C tachypnea at 45 breaths per minute, normal heart rate closer to 100 beats per minute. Her oxygen saturation was 98% at room air. Her body weight was 10 kilograms, and her size was 88 cm. The pleuropulmonary examination found signs of respiratory distress, such as intercostal, subcostal, and supraclavicular retractions and decrease in vocal resonance. On auscultation, there was absence of breath sounds and tympanism in the right chest. The chest X-ray showed a right pneumothorax (Figure 1) that was drained. The laboratory data showed leukocytosis at 16,000/mm^3^, predominantly neutrophilic at 8,360/mm^3^, hemoglobin at 7.9 g/dl, and CRP at 70 mg/dl, and the blood culture was sterile. The patient was treated by amoxicillin-clavulanic acid without much clinical and radiological improvement. A thoracic CT scan searching an underlying pathology revealed overdistension of the right hemithorax with parenchymal air cystic lesions involving the external segment of the middle lobe conducting to a malformation cystic adenomatoid, a fairly abundant right hydropneumothorax responsible for a compressive effect on the adjacent pulmonary parenchyma, and inflammation of the pleura (Figure 2). The patient has been scheduled for surgery; however, she was lost because of COVID-19 pandemic and social distancing, so she missed her scheduled surgery. Two months later, she presented with pyopneumothorax that was drained. The drain brought back 100 ml of yellow ladle liquid with lumps of pus. Bacteriological examination of pus isolated *Pseudomonas aeruginosa* and *Klebsiella pneumoniae*. A right middle lobectomy was performed because we suspected a cystic adenomatoid malformation of the right middle lobe. Through an open thoracotomy, we found a gelatinous component in the right chest originated from the middle right lobe extending to the pleura and diaphragm; a middle lobectomy was performed with resection of the gelatinous component (Figure 3).The specimen weighed 194 g and measured 16 cm. Cytological finding confirmed macroscopically multiple fragments with polypoid cerebroid and necrotic component and rare cystic wall.Microscopically, the tumor proliferation had the double epithelial (Figure 4) and sarcomatous component (Figure 5). We conducted type II of pneumoblastoma. The extension assessment found secondary hepatic localization. Adjuvant chemotherapy has been indicated in addition to surgery, and the child was referred to the pediatric oncology unit. Six months after the surgery, the patient developed a local recurrence that is still being treating by chemotherapy.

## 3. Discussion

Pulmonary blastoma is an uncommon tumor. This rare and very aggressive tumor has unspecific clinical and radiological characteristics which often delay the diagnosis. In general, respiratory symptoms such as cough, chest pain, dyspnea, and hemoptysis are most common in PB patients. Other clinical presentations, including pneumothorax, fever, hemothorax, and pleural effusion, may also be encountered [4]. Chen et al. reported a case of a large pulmonary blastoma in a 7-year-old girl whose initial presentation was progressive dyspnea and productive cough [5], which was similar to our case who had cough dyspnea, and the pulmonary blastoma was revealed by pneumothorax. In fact, radiological images are being often confused with those of a congenital lung defect. Regarding the localization, the right lung is more frequently affected by pulmonary blastoma than the left, which is consistent with a previous study involving adult patients [6]. From a cytological point of view, it is an embryonal tumor of the lung with epithelial and mesenchymal components. The solid regions in types II and III were histologically similar, displaying a mixed, sarcomatous pattern. These sarcomatous cells may include interspersed foci of anaplasia, features of embryonal rhabdomyosarcoma, chondrosarcoma, or necrosis [7–9]. PB types 1 and 2 had areas very similar to congenital pulmonary airway malformation type 4; however, the latter has no immature/malignant components. Cytogenetically, these are completely different lesions.

Our patient had large foci of tumor necrosis. In fact, the biologic behavior of the tumor was unpredictable. However, no sensitive and specific serum tumor marker was available for its diagnosis.

Formerly, controversy existed regarding whether a pulmonary malformation could degenerate into a PB [10–12]. However, detailed genetic and immunohistochemical explorations have confirmed that distinct pathogenic mechanisms exist between these two disease entities [13, 14]. In fine, we concluded that the pathology is mandatory to confirm pulmonary blastoma diagnosis. Knight et al. reported that types II and III may metastasize most often to the brain but rarely to the liver and bone [15], which contrasts with our patient because she metastasized to the liver. Overall, our management was surgery with chemotherapy.

The European cooperative study group for pediatric rare tumors has children with a threshold of tumor's size, and tumors were large at 10 cm in maximum diameter as a reasonable cutoff point, above which up-front biopsy followed by neoadjuvant chemotherapy is a reasonable approach [16]. In our case, neoadjuvant chemotherapy was used in order to minimize the radical surgical approach needed to achieve local control.

Surgical resection of type II and type III PPB may require a wedge resection lobectomy or pneumonectomy to achieve negative margins if possible. When performing a lobectomy for type II or III PPB, involved pleural surfaces should be resected with the primary tumor and involved pulmonary lobe. For large type II or III PPB with extensive pleural spread, an extrapleural pneumonectomy may be required to achieve local control [17]. In our case, the surgery was not initially carried out for cancer purposes, so neoadjuvant chemotherapy was used in order to minimize the radical surgical approach needed to achieve local control. In addition, we emphasize the importance of completing surgical excision because it appears as an important prognosis factor.

Significant correlation occurred between the PPB type and survival which is type I at 94%; type II at 71%, and type III at 53% [8]. In our case, the factors of bad prognosis were the sarcomatous component with local recurrence and hepatic metastasis.

Finally, pulmonary blastoma is a rare tumor which must be in mind even if we have no specific signs. Therefore, we have to think about in front of a pulmonary malformation and also a pneumothorax. A few cases have been reported presenting as a spontaneous pneumothorax, even in the absence of any demonstrable solid mass [18, 19]. Further studies focusing on the pathway to this rare disease are mandatory to provide new treatments' insights.

## 4. Conclusion

Pneumoblastoma in children is a rare, aggressive tumor that shows up with nonspecific clinical and radiological signs which may often delay diagnosis, so the prognosis may be so bad. Pathological confirmation is mandatory.
